# Using Participatory Action Research to Enhance Physical Education Interventions for Promoting Active Lifestyles in Schools: A Study Design and Protocol

**DOI:** 10.3390/healthcare13182362

**Published:** 2025-09-19

**Authors:** Jorge Lizandra, Alexandra Valencia-Peris, Roberto Ferriz, Carmen Peiró-Velert

**Affiliations:** Department of Teaching Physical Education, Arts and Music, Faculty of Teacher Training, Universitat de València, 46022 València, Spain; alexandra.valencia@uv.es (A.V.-P.); roberto.ferriz@uv.es (R.F.); carmen.peiro@uv.es (C.P.-V.)

**Keywords:** youth engagement, health promotion, collaborative methodologies, self-determination theory, salutogenic approach

## Abstract

Promoting active lifestyles among adolescents is essential due to their short-, medium-, and long-term contributions to young people’s holistic development and overall health. Beyond physical well-being, Physical Education foster physical activity, autonomy, social connectedness, motivation and emotional well-being, thus constituting a key dimension of quality education. Background/Objectives: The “Estilos de Vida Activos (EVA)” project is a school-based intervention designed to foster adolescent agency and motivation in adopting active habits. Grounded in the salutogenic model, self-determination theory, and the health-based Physical Education pedagogical model, this protocol describes the design and implementation strategies of a participatory intervention in secondary schools. Methods: A variety of research methods will be used to collect quantitative and qualitative data before, during, and after the intervention. Validated questionnaires will assess active commuting, socioeconomic status, satisfaction of basic psychological needs, motivation, levels and intention to engage in physical activity. Qualitative data include interviews with teachers, Photovoice sessions with students, observation notes, and programme materials. Intervention: The EVA intervention is collaboratively developed by students, teachers, and researchers using participatory action research. It includes needs analysis, participatory activities, and co-design of tailored physical activity programmes. The intervention is described using the Template for Intervention Description and Replication checklist (TIDieR) to enhance transparency and replicability. Conclusions: This protocol presents a theoretically grounded and participatory approach to school-based health promotion. By integrating educational and collaborative strategies, it offers a replicable model that promotes adolescent active lifestyles, from contextual relevance, and pedagogical coherence, serving as a guide for inclusive and sustainable interventions in school settings.

## 1. Introduction

Despite the well-documented benefits of physical activity (PA), a significant proportion of adolescents remain physically inactive. Globally, over 80% of adolescents fail to meet the World Health Organisation (WHO) PA recommendation of at least 60 min of moderate-to-vigorous physical activity per day [1], and in Spain the situation is comparable, particularly among girls and older adolescents [2]. This public health concern has prompted the WHO’s Global Action Plan on PA 2018–2030, calling for coordinated, multisectoral interventions to increase PA participation [3].

Schools are widely recognised as ideal environments for promoting PA behaviours due to their broad reach, structured routines, and influence from key social agents such as teachers, peers, and families [4]. Within these settings, Physical Education (PE) plays a key role in developing health-related knowledge, attitudes, and behaviours [5]. However, most school-based interventions have been designed within a biomedical paradigm that emphasises behaviour modification through prescriptive and often top-down methodologies [6,7]. Although some have reported short-term increases in PA [8,9,10], their long-term effectiveness and behavioural transfer to adolescents’ daily lives remain questionable [11]. In this sense, the salutogenic perspective emerges as a more appropriate approach as it shifts the focus from disease prevention to the identification and mobilisation of individual and environmental resources that support health and well-being [12,13].

This lack of sustained impact may also be explained by the limited involvement of adolescents in the design and implementation of these interventions. Many continue to treat students as passive recipients rather than active contributors, resulting in programmes misaligned with their lived experiences, motivations, and contexts [14]. In contrast, participatory and asset-based approaches seek to empower youth by recognising their strengths, needs, and aspirations. These approaches privilege youth perspectives, grounded in children’s rights discourses—particularly Article 12 of the United Nations Convention on the Rights of the Child (United Nations, 1989)—which affirms their right to express views on matters affecting them [15,16]. According to Sandford et al. (2023) [17], voice can be understood as how youth are able to communicate their thoughts and feelings and, in doing so, play an active role in society. This voice refers not only to verbal expression but also to diverse communicative forms, including art, writing, and even silence, acknowledging the varied ways youth articulate their experiences [18,19].

Then, schools aiming at promoting healthy lifestyles need to find strategies to involve students to discover their perspectives and to empower them for action. On one hand, it will respect their agency, i.e., active subjects, in understanding and influencing their behaviours and school context [20]. On the other hand, students are the real experts on their own lives and contexts, and their perspectives will provide pivotal information to design health promotion interventions [21]. In this sense, methods aimed to stimulate dialogue and to build shared meanings among equals are needed for enhancing self-driven learning process among children and adolescents [22].

From this perspective, the salutogenic and positive approach to health development prioritises the creation of conditions that enhance personal growth, agency, and meaningful engagement in health-promoting activities, rather than focusing solely on the reduction in risk behaviours. In the context of adolescent health, this perspective underscores the importance of strengthening protective resources—such as resilience, perceived competence, and social connectedness—which are central to supporting the adoption and maintenance of active and healthy lifestyles [22,23]. This orientation aligns closely with contemporary pedagogical models in PE—such as health-based Physical Education (HBPE) [24]—which draw on the principles of Self-Determination Theory (SDT). Within adolescence, SDT provides a particularly valuable framework as it highlights the role of autonomy, competence, and relatedness in fostering intrinsic motivation and sustained behaviour change [25]. Recent contributions have also emphasised novelty as an additional psychological need that may be especially relevant during adolescence, a developmental stage characterised by the search for new and stimulating experiences [26]. Together, these theoretical perspectives inform educational approaches that seek to create enjoyable, meaningful, and transferable learning experiences, thereby fostering health-promoting behaviours within and beyond the school context [27]. In this sense, participatory action research (PAR) methodologies, such as Structured Interview Matrix (SIM) and Photovoice, have shown promise in enhancing student agency, generating contextually grounded knowledge, and co-creating interventions that are meaningful and feasible [28,29,30]. These strategies have been successfully applied in school-based studies with adolescents [22,23], where they have supported the development of positive, realistic, and sustainable health practices by encouraging students to identify assets, share narratives, and reflect collectively [31].

This article presents the protocol of the EVA (*Estilos de Vida Activos*; Active Lifestyles) project, a school-based pilot intervention co-designed and implemented with students and teachers in Spanish secondary schools. EVA was based on the SALVO project (Stimulating Active Lifestyles in Vocational Training), an initiative originally developed in the Netherlands to promote student agency in PA through participatory methodologies in secondary vocational education settings [32]. SALVO emphasises the importance of empowering students to shape meaningful PA experiences by recognising their personal assets and fostering autonomy. The EVA project adapts these principles to the Spanish educational context, integrating participatory action research (PAR) strategies within the PE curriculum to foster adolescents’ active participation in the promotion of healthy behaviours. Therefore, the central research question guiding this study is as follows: *How can the participatory identification of health assets contribute to the design of school-based interventions that support adolescents in adopting, maintaining, and committing to more active and healthier lifestyles?* Specifically, the objectives of the EVA project are (i) to support teachers and students in identifying and mobilising the health assets available in their contexts that facilitate the adoption of active and healthy lifestyles; (ii) to accompany participants in the planning, development, and evaluation of PA and health programmes from a participatory perspective; and (iii) to explore which elements participants consider transferable to their daily lives. Given the limited application of such approaches within curricular PE, this study could offer a relevant contribution by aligning with current international evidence-based recommendations on school-based health promotion policies [33].

## 2. Materials and Methods

### 2.1. Study Context and Participants

The EVA project was implemented in two public secondary schools located in the Region of Valencia, Spain, as part of the PE curriculum during the 2020–2021 and 2021–2022 academic years. Participants included 130 students (aged 13 to 16), four PE teachers, and the two school management teams. The intervention was integrated into regular PE classes and approved by the school councils. Schools were selected through convenience sampling, following a prior teacher training programme in which several schools had participated. Two schools were chosen based on their willingness to engage more intensively in the project and the research team’s capacity to provide close follow-up, with the additional criterion of including one school located in a large urban area and another in a smaller municipality.

### 2.2. Overall Study Procedure

**Figure 1:**
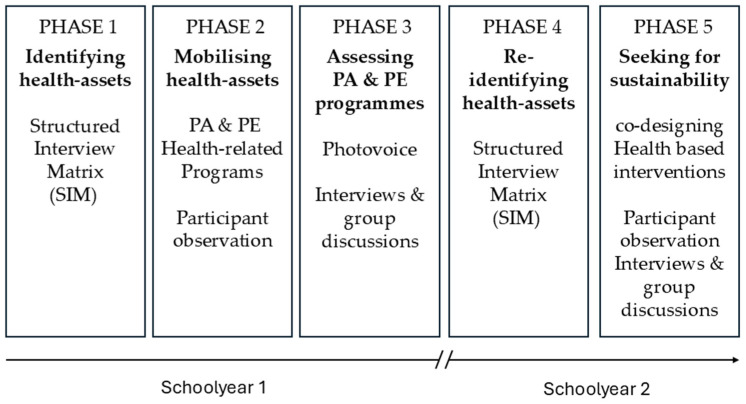
EVA project intervention phases.

The intervention was developed collaboratively between the research team and the participating schools from October 2020 to May 2022 and included five main phases (see Figure 1). It comprised a series of co-designed, school-based activities aimed at promoting healthy lifestyles through active and participatory pedagogical strategies. Specifically, collaboration was embedded throughout the process and included (i) obtaining consent and offering a structured training programme, as well as several preparatory meetings to tailor the intervention to the context of each school, prior to implementation; (ii) jointly identifying health assets through SIM sessions; (iii) co-designing and implementing PA and health-related PE programmes; (iv) evaluating these programmes using PAR strategies; and (v) maintaining an ongoing dialogue between researchers, teachers and students through meetings, interviews and group discussions.

### 2.3. Research Design

To ensure a transparent, systematic and replicable description of the intervention, the Template for Intervention Description and Replication checklist (TIDieR) [34] was employed (Table 1). This reporting guideline was specifically designed to improve the quality of intervention protocols, particularly in complex and context-dependent settings such as schools [35,36]. Unlike frameworks such as the Replicating Effective Programmes Framework (REP) [37], which focus on the adaptation and implementation of previously validated programmes, TIDieR allows for a comprehensive description of novel interventions during their design and pilot phases [38]. It also serves as a structured guide to describe and implement the intervention components and was selected due to its suitability for participatory educational interventions that are still in the pilot phase of development. Therefore, it was considered the most appropriate framework to structure the methodology of the EVA project.

### 2.4. Research Instruments and Procedures

A variety of methods will be employed to gather data before, during, and after the intervention. Quantitative data will be collected at two time points: baseline (one week before the intervention) and post-intervention (one week after its completion) through validated questionnaires. All student participants will complete the questionnaires; teachers will not participate in this component. Specifically, basic psychological needs will be assessed using the Basic Psychological Needs in Exercise Scale (BPNES), adapted to Physical Education [40], and complemented with items from the Novelty Need Satisfaction Scale (NNSS) [25]. Motivational regulation will be measured using the Spanish version of the Perceived Locus of Causality Scale (PLOC) [41], while participants’ intention to engage in future PA will be assessed through an ad hoc 3-item questionnaire developed by the research team. PA levels will be assessed using the Spanish short version of the International Physical Activity Questionnaire (IPAQ) [42], and active commuting habits will be evaluated through the Mode of Commuting to and from School Questionnaire [43]. Socioeconomic status will be determined through the Family Affluence Scale III (FAS III) [44]. Response scales varied across instruments, ranging from 5-point Likert scales (e.g., for BPNES and PLOC) to categorical frequency-based options (e.g., for IPAQ and commuting habits) (see Supplementary File).

Qualitative data will be collected using a range of complementary strategies to ensure methodological triangulation across data sources and participant groups, thereby enhancing the credibility and depth of the findings [45,46]. These will include semi-structured interviews with PE teachers conducted before and after the intervention, field notes recorded by researchers and facilitators during programme implementation, and group discussions with students following the Photovoice sessions. Interview and focus group guides will be structured around key themes including perceptions of health and PA, barriers and facilitators to engagement, experiences with the co-designed programmes and reflections on learning and empowerment. In addition, documentary materials—particularly the student portfolios and pedagogical tools developed throughout the intervention—will be analysed as artefacts to understand the learning processes and the relevance of the co-designed PA and health PE programmes.

### 2.5. Data Analysis Plan

Quantitative data will be analysed with SPSS version 28 (IBM, New York, NY, USA). Descriptive statistics will be used to summarise baseline characteristics and outcome variables. To assess changes between pre- and post-intervention measures, inferential tests such as repeated measures tests or their non-parametric equivalents will be applied, depending on data distribution. Analyses will be conducted separately for each participating school, and where appropriate, comparisons between schools will be explored to identify contextual differences. These analyses are aligned with the project’s objectives, particularly in evaluating the impact of co-designed programmes on students’ motivation, PA levels, and perceived transferability of learnings.

Qualitative data from SIM method, Photovoice murals, individual interviews and group discussions will be analysed using thematic analysis, following Braun and Clarke’s approach [47]. Coding will be performed inductively through NVIVO version 14. Emerging themes will be developed through iterative cycles of coding and discussion among the research team and contrasted with participants to enhance credibility. Given that the two schools implemented the EVA intervention in idiosyncratic ways, the study will employ a cross-case comparison to identify specific units of analysis and systematically explore similarities, differences, and emerging patterns. Following Stake’s approach to case study research [46], this comparative analysis will include a vertical dimension in which the main features, contexts and processes of each case are examined in detail. This approach is particularly useful for understanding how contextual conditions, as well as teacher–student interactions and decision-making, shape the development and outcomes of the EVA intervention, thereby explaining variations in strengths and weaknesses between the two schools. To ensure the rigour of the research, strategies such as prolonged engagement, member checking, peer debriefing and methodological triangulation will be embedded throughout the study design and implementation.

### 2.6. Ethical Considerations

The study protocol was reviewed and approved by the Ethics Committee of the Universitat de València (UV-INV_ETICA-1189475). All participants (schools’ staff, students and their families) received detailed information about the study and provided written informed consent. Anonymity and confidentiality were guaranteed throughout the research process, and the intervention was aligned with the principles of equity, inclusion and voluntary participation. The participatory methods used in the EVA project were implemented with attention to power dynamics and the active involvement of students in shaping the process. In this regard, as mentioned above, students play a central role in the intervention and actively participate in all phases of the project: identifying health assets in their school and community, prioritising which assets to mobilise, co-designing and implementing physical activity and health programmes tailored to their needs, and reflecting on the transferability of acquired learning to their daily lives.

## 3. Discussion

### 3.1. Contextualising the EVA Project Intervention: Adaptation and Complementarity with the SALVO Project

The EVA project draws its foundational structure and inspiration from the Dutch SALVO initiative, a school-based health promotion intervention grounded in the salutogenic approach and PAR strategies such as the SIM and Photovoice [22]. The replication of effective interventions in different settings requires achieving a balance between fidelity to core elements and contextual adaptation [48,49]. In the case of EVA, fidelity was ensured by preserving the intervention’s participatory logic, health asset-based assessment and integration within the school community.

However, significant adaptations were necessary to integrate the programme into the Spanish educational context. Whereas SALVO operated at a whole-school level, EVA was designed to be embedded within the PE curriculum. This choice responds to the reality that in Spain—and particularly in the Valencian region—health promotion efforts in schools are still predominantly led by PE departments [50,51]. Starting within PE allowed the intervention to capitalise on existing structures and professional expertise while maintaining potential for future school-wide expansion. Furthermore, the use of PAR strategies was also adapted. While SIM was retained for identifying health assets, Photovoice was reconceptualised as a reflective evaluation tool during the implementation phase, allowing students to document and assess their own learning and behavioural changes. This use of visual and narrative inquiry aligns with the pedagogical potential of portfolios as tools for student reflection and engagement [32,52].

Overall, this flexible and context-sensitive approach reinforces the replicability of EVA in similar settings, supporting the idea that sustainable health promotion interventions require both fidelity to essential components and adaptive integration within specific cultural and curricular frameworks [48,49].

### 3.2. Methodological Contributions: Participatory Approaches in Educational Health Promotion

One of the most distinctive contributions of the EVA protocol lies in its use of participatory strategies that give voice to students and teachers as co-creators of health-promoting environments. Traditional health interventions have often prioritised biomedical frameworks; however, alternative approaches increasingly emphasise student agency and the co-construction of meaningful learning experiences. For instance, Whitley et al. [53] highlighted how co-creation with marginalised youth in community sport-for-development programmes facilitated not only participation but also empowerment and positive identity formation. In school contexts, Casey and Goodyear [54] demonstrated how pedagogical models that emphasise student agency and the co-construction of learning tasks can foster more autonomous and active identities. Complementing these approaches, Bachouri-Muniesa et al. [23] developed a multilevel, multicomponent school-based intervention through a stakeholder-driven co-creative process, engaging teachers, families, and policymakers to enhance contextual adaptation, ownership, and sustainability. Collectively, these studies illustrate the growing importance of co-creation as a pedagogical and methodological principle in designing interventions that aim to nurture active identities in youth. In this sense, EVA aligns with this evolving paradigm by fostering collaboration and shared responsibility among educational stakeholders.

In particular, the EVA project is underpinned by pedagogical models such as HBPE [24,55], which integrates the principles of SDT [25] to create motivational climates that support autonomy, competence, relatedness and novelty, four key psychological needs that underpin intrinsic motivation and long-term behavioural adherence [26]. Therefore, these models not only position health as a holistic construct embedded in the school curriculum, linking physical activity with students lived experiences and cultural contexts, but also provide a pedagogical framework for transforming PE into a meaningful, student-centred learning space. In fact, this approach is not only consistent with international health promotion frameworks [33] but may also be regarded as a precursory initiative to the most recent Spanish guideline on Health-Promoting Schools [56].

By involving students in identifying health assets and co-designing action plans, EVA not only promotes engagement and ownership but also enhances contextual relevance and cultural appropriateness. As supported by the recent literature, participatory methodologies are essential for generating long-term behavioural changes and building sustainable school health initiatives [57,58]. Furthermore, the integration of SIM and Photovoice supports deeper understanding of students’ perceptions and motivations, offering valuable insights for educators and policymakers alike. Based on a previous study [32], both methods not only provided richer qualitative data but also fostered active student agency by creating safe and structured spaces, both within the school and in community settings, where adolescents could articulate their needs, values and experiences. Moreover, amplifying students’ voices empowers them to co-construct meaningful health-related knowledge, thereby strengthening the relevance and transferability of school-based interventions.

### 3.3. Potential of a Plurality of Research Method in School-Based Interventions

The adoption of a variety of research methods represents another strength of the EVA protocol. While quantitative data enable the measurement of behavioural changes, motivational profiles, and socioeconomic differences across diverse student groups, qualitative data provide contextual depth and explanatory power. This methodological complementarity allows for a more holistic understanding of how and why interventions succeed or fail in different settings [59,60].

The process evaluation component, incorporating students’ portfolios, teachers’ interviews, and observational data, serves not only to assess the implementation fidelity but also to capture the dynamic nature of behaviour change in adolescence. Moreover, the triangulation of data sources and informants—teachers, students and researchers—enhances the validity and credibility of findings and supports the transferability of the intervention to other educational contexts [47,61].

Nonetheless, future research directions could focus on the implementation of mixed-methods approaches which, as highlighted in the literature, not only enhance the rigour and adaptability of interventions but are particularly valuable for developing multicomponent, multilevel and context-sensitive strategies aimed at addressing the multifactorial nature of adolescent health behaviours [62,63].

## 4. Conclusions

This article has presented the protocol of the EVA project, a school-based intervention grounded in a salutogenic perspective and designed through active and participatory methodologies. By embedding health promotion within the PE curriculum, the project offers a context-sensitive and pedagogically sound approach that enhances student engagement and agency in promoting active lifestyles.

Beyond its methodological robustness, EVA represents a coherent and potentially replicable model of intervention tailored to the challenges of health promotion in secondary education. Its emphasis on student voice, interdisciplinary collaboration, and iterative development underscores its relevance for both educational practice and public health research.

At the same time, participatory methodologies also involve practical challenges, such as limited teacher availability, resistance to innovation, and fluctuating levels of student engagement. Recognising these constraints is key to strengthening the feasibility and impact of future interventions. Moreover, combining quantitative and qualitative approaches provides a comprehensive understanding of how such programmes operate and how they can be adapted to diverse educational contexts.

In sum, the EVA protocol offers a theoretically grounded and contextually adaptable framework for fostering active lifestyles in schools. Future research will evaluate its effectiveness and capacity to promote meaningful and sustainable behavioural change among adolescents, with the ultimate goal of informing broader educational and health policy initiatives.

## Figures and Tables

**Table 1:** TIDieR checklist for the description and replication of the EVA project intervention (adapted from Hoffmann et al., 2014 [34]).

Brief name	EVA Project: An intervention for promoting active lifestyles in schools from PE lessons.
2. Why?	- Because of the need to develop participatory and collaborative interventions to challenge research proposals that often treat participants as passive subjects [14]. - To tackle the alarming data on PA practice. Approximately 80% of adolescents fail to meet PA recommendations [3], with this figure being particularly alarming among girls and showing a decreasing trend as young people approach or reach adulthood. - To engage students and thus connect with their interests and needs. PAR strategies are particularly suitable models for developing interventions that aim to achieve the desired transfer in adolescents’ active behaviour during their leisure time. - The intervention proposal is grounded in the salutogenic approach to health from the model of social and individual determinants of health [39], as well as in the SDT [25].
3. What? Materials	- Colour stickers for organising work groups (red, yellow, green, blue). - Work sheets for SIM and Photovoice dynamics. - 4 audio recorders (for SIM, Photovoice, and the development of interviews and discussion groups). - PowerPoint presentation outlining the phases of SIM, the PA and health PE programme grounded on the HBPE [24]. - 8 white continuous paper murals (4 for SIM and 4 for Photovoice) and coloured markers. - Curricular materials for programme development (contract, FITT-PV (Frequency, Intensity, Time, Type of Activity, Progression and Variety) programming sheets, illustrated portfolio, routine of thoughts for session explanation and development). - Questionnaires for assessing changes in the health-related variables (PA, sedentary behaviour, active commuting, motivation and intention of future practise of PA). (See Research Instruments and Procedures section). - EVA project website where all work materials can be accessed. (www.projecteeva.es; accessed on 1 September 2020).
4. What? Procedures	- The intervention is included as part of the didactic programme for the PE subject. - Before starting, consent is obtained from the school management team, PE teachers, students and families. - Identifying health assets through SIM [22]: Two 45 min sessions are conducted. In the first session, groups are organised, initial interviews related to the four facilitating elements of active lifestyle configurations (passions, social environment, physical environment and skills) are prepared and six rounds of interviews (1 × 1) are performed. In the second session, each group shares the information, prepares the explanatory mural and concludes with a plenary presentation. - Mobilising health assets (PA and health-related PE programmes): Each group selects 1 or 2 health assets and designs a 6-week programme with a minimum of 2 weekly sessions devoted during PE classes and 1 extracurricular session. They should take photos of the process and collect all the evidence in the portfolio. - Assessing PE Programmes through Photovoice [22]: Two 45 min sessions are conducted. In the first session, group discussions are held regarding the images selected by each participant and their relation to the four facilitating elements of active lifestyle. In the second session, similar to SIM, each group shares the information, prepares the explanatory mural, and concludes with a plenary presentation. - For the intervention study: Interviews are conducted with the teachers before and after the intervention. During the development, meetings are held between the research team and the teachers. Additionally, questionnaires (pre-test/post-test) are administered, and at the end, group discussions are conducted with the students.
5. Who provided?	The research team provides teachers with a prior structured training programme entitled “Design of Educational and Collaborative Interventions for Promoting Active Lifestyles in PE Classes,” consisting of 18 to 30 h of training. The following topics are covered: - Concept of health based on positive health and the model of health determinants. - HBPE pedagogical model. - Motivational strategies to support autonomy, competence, relatedness and novelty to develop intervention programmes aimed at promoting active lifestyles. - SIM and Photovoice as strategies for mapping and mobilising health assets. - Designing intervention and curriculum materials adapted to different teaching contexts. Subsequently, all phases of the intervention are prepared with the teachers. In addition to the research team, teachers and students, the presence of a facilitator is crucial to energise the workgroups. The facilitator can be a member of the research team or university or school students who have been previously trained for this role. Training consisted of explaining the functioning of SIM and Photovoice and illustrating facilitator actions from previous projects.
6. How?	The intervention is conducted in-person during PE classes and some sessions during tutorial periods. The intervention is implemented in various class groups. The extracurricular sessions are monitored for PE teachers by the portfolio. The working team consists of the PE teachers and 2–4 members of the research team (depending on whether they take on the role of facilitators).
7. Where?	Most of the intervention is carried out within the facilities of the school. The extracurricular sessions could be wherever students decided depending on the contents and aims of the PA and heath PE programme.
8. When and how much?	The whole intervention of the EVA project has been performed, up to this point, in two high schools. The pre-training for teachers takes around two months. All the activities described in Section 4 of this checklist took place from January to May 2021 (School Year 1) and from October to May 2022 (School Year 2), with the following temporal distribution: October–November 2020: Pre-training for the teaching staff and preparation of materials. January: Planning and organisation of the intervention. February–April 2021: Commencement of the SIM (2 sessions) and the PA and health-related PE programme (3 weeks for designing and providing feedback, 6 weeks of implementation). Parallelly, conducting interviews and meetings with the teaching staff. May 2021: Conducting the evaluation trough Photovoice (2 sessions) and carrying out the group discussions with the students. Closure of the intervention. October 2021: Re-identifying health assets with 2nd SIM for developing sustainable health-based interventions. November 2021–May 2022: Co-designing, participant observation and assessment of the sustainable health-based interventions.
9. Tailoring	The intervention is adapted to the schedules and routines of the schools. In the original proposal of the SALVO project, SIM and Photovoice were conducted in single sessions lasting between 90 and 120 min. The integration of the intervention into the PE subject, as well as the incorporation of the HBPE pedagogical model, are adaptations made by the EVA project in relation to the original proposal.
10. Modifications	Initially, we considered allowing students to include any health assets they desired in the PA and health PE programme. However, to facilitate the PE teachers’ ability to track the programme’s content, we subsequently decided to restrict it to two health assets. Furthermore, we incorporated the illustrated portfolio and the reflection routine to assist students in comprehending the concepts of the PA and health-related PE. Although these curriculum materials were not part of the original proposal, they were provided to students during the phase of programme design.
11. How well? Planned (fidelity)	Since this is the first implementation of the intervention, fidelity assessment is not initially considered. However, the steps and structure of the SIM and Photovoice, although adapted to the school context, are developed following the same methodology learned from the SALVO Project. The research group members are responsible for ensuring fidelity to these methods.
12. How well? Actual	The final design of the intervention was effectively implemented in both school contexts. We acknowledged and embraced certain individual and between-group differences in the structure and implementation of the PA and health-related PE (i.e., differences in health assets identified, in the procedures followed to mobilise them, and in the strategies adopted according to the characteristics of each school context, the teachers guiding the process, and the educational community involved). This aspect of the intervention allowed for creativity and recognised that variations are inherent to the pedagogical approach and the need for individual and contextual adaptation in each specific case.

## Data Availability

The data and procedures supporting the reported results are available upon request to the corresponding author.

## Supplementary Materials

The following supporting information can be downloaded at https://www.mdpi.com/article/10.3390/healthcare13182362/s1, File S1. EVA project research instruments.

## Abbreviations

The following abbreviations are used in this manuscript:

WHO	World Health Organisation
PA	Physical Activity
PE	Physical Education
HBPE	Health-Based Physical Education
SDT	Self-Determination Theory
SIM	Structured Interview Matrix
PV	Photovoice
EVA	Estilos de Vida Activos (Active lifestyles)
PAR	Participatory Action Research
TIDieR	Template for Intervention Description and Replication
FITTPV	Frequency, Intensity, Time, Type of Activity, Progression and Variety
SALVO	Stimulating Active Lifestyles in Vocational Training
IPAQ	International Physical Activity Questionnaire
PLOC	Perceived Locus of Causality Scale
BPNES	Basic Psychological Needs in Exercise Scale
NNSS	Novelty Need Satisfaction Scale
FAS III	Family Affluence Scale III
