# Supplementary material for: Using Participatory Action Research to Enhance Physical Education Interventions for Promoting Active Lifestyles in Schools: A Study Design and Protocol
Source: Healthcare (Basel). 2025 Sep 19;13(18):2362. doi: 10.3390/healthcare13182362 (PMC12469524; doi:10.3390/healthcare13182362)
Supplement: Supplementary file 1 [file healthcare-13-02362-s001.zip › healthcare-3816590-supplementary.pdf]

CÓDIGO IDENTIFICACIÓN: | | | | | | | |

# CUESTIONARIOS DEL PROYECTO *"EVA PROJECT: UNA INTERVENCIÓN EDUCATIVA Y COLABORATIVA PARA LA PROMOCIÓN DE ESTILOS DE VIDA ACTIVOS DESDE LA EDUCACIÓN FÍSICA"*

El grupo de investigación *Actividad Física, Educación y Sociedad (AFES)*, del Departamento de Didáctica de la Expresión Musical, Plástica y Corporal de la Universitat de València estamos realizando un estudio de intervención en centros educativos para la promoción de estilos de vida activos desde la asignatura de Educación Física. Una parte de esta investigación consiste en cumplimentar los siguientes cuestionarios, para lo que necesitamos tu colaboración. Puedes responder con toda sinceridad a las preguntas, pues se trata de un documento anónimo y de carácter confidencial.

**GRACIAS por tu colaboración en este estudio.**

**DATOS PERSONALES**:

**Edad**: 1. Hombre ☐ 2. Mujer ☐ 3. Otro ☐

**País de nacimiento**: ________________ **Localidad actual**: __________________

**Estatura** ☐ **Peso** ☐

**1. ¿Tiene tu familia coche propio u otro vehículo motorizado (furgoneta, moto…)?**

0. ☐ No 1. ☐ Sí, uno 2. ☐ Sí, dos o más

**2. ¿Tienes tu propio dormitorio?**

0. ☐ No 1. ☐ Sí

**3. ¿Cuántos ordenadores tiene tu familia? (incluyendo portátiles y tablets, NO incluyendo videoconsolas y teléfonos móviles)**

0. ☐ Ninguno 1. ☐ Uno 2. ☐ Dos 3. ☐ Más de dos

**4. ¿Cuántos baños (habitación con una bañera/ducha o ambas) hay en tu casa?**

0. ☐ Ninguno 1. ☐ Uno 2. ☐ Dos 3. ☐ Más de dos

**5. Durante los últimos 12 meses, ¿cuántas veces saliste de vacaciones con tu familia? (antes de la COVID-19 y fuera del país)**

0. ☐ Nunca 1. ☐ Una vez 2. ☐ Dos veces 3. ☐ Más de dos veces

**6. Tu familia, ¿Tiene lavavajillas?**

0. ☐ No 1. ☐ Sí

**7. ¿Cómo vas <u>habitualmente</u> al instituto?**

Andando | Bici | Coche | Moto | Bus-escolar | Bus no escolar | Metro/Tren/Tranvía | Otros: ¿Cuál?

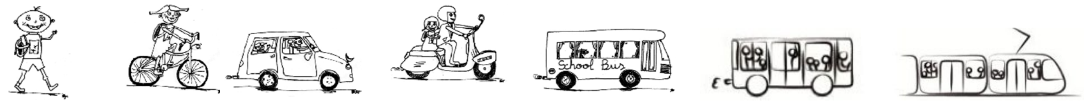


☐ ☐ ☐ ☐ ☐ ☐ ☐ ☐

**8. ¿Cómo vuelves <u>habitualmente</u> del instituto?**

Andando | Bici | Coche | Moto | Bus-escolar | Bus no escolar | Metro/Tren/Tranvía | Otros: ¿Cuál?

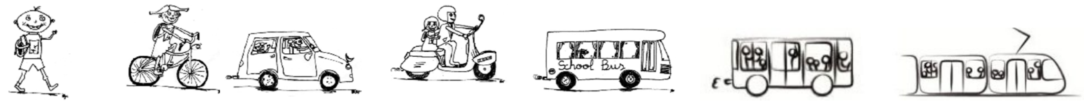


☐ ☐ ☐ ☐ ☐ ☐ ☐ ☐

**9. ¿Cuánto tardas en llegar al instituto, desde que sales de tu casa?**

☐ Menos de 15 minutos ☐ de 15 a 30 minutos ☐ de 30 a 60 minutos ☐ más de 60 minutos

**10. ¿Cuánto tardas en volver a casa, desde que sales del instituto?**

☐ Menos de 15 minutos ☐ de 15 a 30 minutos ☐ de 30 a 60 minutos ☐ más de 60 minutos

**11. ¿Realizas Actividad Física fuera del horario escolar (extraescolar)?**

0. ☐ No. **avanza a la pregunta 14** 1. ☐ Sí

| 11. Cuando realizo ejercicio físico o deporte (fuera de las clases de Educación Física)… | Totalmente en desacuerdo | En desacuerdo | Neutro | De acuerdo | Totalmente de acuerdo |
|---|---|---|---|---|---|
| 1. Siento que hago cosas novedosas | 1 | 2 | 3 | 4 | 5 |
| 2. El ejercicio físico o deporte que realizo está muy relacionado con lo que me gusta y me interesa | 1 | 2 | 3 | 4 | 5 |
| 3. Creo que he progresado enormemente con respecto al objetivo final que persigo | 1 | 2 | 3 | 4 | 5 |
| 4. Me siento muy cómodo con mis compañeros de ejercicio físico o deporte | 1 | 2 | 3 | 4 | 5 |
| 5. Siento que me proporciona experiencias novedosas | 1 | 2 | 3 | 4 | 5 |
| 6. Estoy convencido/a de que el ejercicio físico o deporte que hago se ajusta perfectamente a la manera en la que prefiero hacerlo. | 1 | 2 | 3 | 4 | 5 |
| 7. Creo que realizo con gran eficacia los ejercicios de mi deporte o ejercicio físico | 1 | 2 | 3 | 4 | 5 |
| 8. Creo que me relaciono con mis compañeros/as de ejercicio físico o deporte de forma muy amistosa. | 1 | 2 | 3 | 4 | 5 |
| 9. Experimento sensaciones nuevas | 1 | 2 | 3 | 4 | 5 |
| 10. Creo que la forma que tengo de hacer ejercicio físico o deporte responde a mis deseos | 1 | 2 | 3 | 4 | 5 |
| 11. Creo que el ejercicio físico o deporte es una actividad que hago muy bien | 1 | 2 | 3 | 4 | 5 |
| 12. Creo que puedo comunicarme fácilmente con mis compañeras/os de ejercicio físico o deporte | 1 | 2 | 3 | 4 | 5 |
| 13. Creo que se plantean situaciones novedosas para mí | 1 | 2 | 3 | 4 | 5 |
| 14. Creo fuertemente que tengo la oportunidad de tomar decisiones respecto a la manera en que hago ejercicio físico o deporte | 1 | 2 | 3 | 4 | 5 |
| 15. Creo que soy capaz de cumplir las exigencias del programa de ejercicio físico o deporte que sigo. | 1 | 2 | 3 | 4 | 5 |
| 16. Me encuentro muy a gusto con mis compañeras /os de ejercicio físico o deporte | 1 | 2 | 3 | 4 | 5 |
| 17. Tengo la oportunidad de innovar | 1 | 2 | 3 | 4 | 5 |
| 18. Creo que descubro cosas nuevas a menudo | 1 | 2 | 3 | 4 | 5 |

**CUESTIONARIOS EN EL CONTEXTO DE LA AF EXTRACURRICULAR**

| 12. Yo hago ejercicio físico o deporte (fuera de las clases de Educación Física)… | Totalmente en desacuerdo | En desacuerdo | Neutro | De acuerdo | Totalmente de acuerdo |
|---|---|---|---|---|---|
| 1. Porque los demás me dicen que debo hacerlo | 0 | 1 | 2 | 3 | 4 |
| 2. Porque me siento culpable cuando no lo practico | 0 | 1 | 2 | 3 | 4 |
| 3. Porque valoro los beneficios que tiene el ejercicio físico | 0 | 1 | 2 | 3 | 4 |
| 4. Porque creo que el ejercicio es divertido | 0 | 1 | 2 | 3 | 4 |
| 5. Porque está de acuerdo con mi forma de vida | 0 | 1 | 2 | 3 | 4 |
| 6. No veo por qué tengo que hacerlo | 0 | 1 | 2 | 3 | 4 |
| 7. Porque mis amigos/familia/pareja me dicen que debo hacerlo | 0 | 1 | 2 | 3 | 4 |
| 8. Porque me siento avergonzado si falto a la sesión | 0 | 1 | 2 | 3 | 4 |
| 9. Porque para mí es importante hacer ejercicio regularmente | 0 | 1 | 2 | 3 | 4 |
| 10. Porque considero que el ejercicio físico forma parte de mí | 0 | 1 | 2 | 3 | 4 |
| 11. No veo por qué tengo que molestarme en hacer ejercicio | 0 | 1 | 2 | 3 | 4 |
| 12. Porque disfruto con las sesiones de ejercicio | 0 | 1 | 2 | 3 | 4 |
| 13. Porque otras personas no estarán contentas conmigo si no hago ejercicio | 0 | 1 | 2 | 3 | 4 |
| 14. No veo el sentido de hacer ejercicio | 0 | 1 | 2 | 3 | 4 |
| 15. Porque veo el ejercicio físico como una parte fundamental de lo que soy | 0 | 1 | 2 | 3 | 4 |
| 16. Porque siento que he fallado cuando no he realizado un rato de ejercicio | 0 | 1 | 2 | 3 | 4 |
| 17. Porque pienso que es importante hacer el esfuerzo de ejercitarse regularmente | 0 | 1 | 2 | 3 | 4 |
| 18. Porque encuentro el ejercicio una actividad agradable | 0 | 1 | 2 | 3 | 4 |
| 19. Porque me siento bajo la presión de mis amigos/familia para realizar ejercicio | 0 | 1 | 2 | 3 | 4 |
| 20. Porque considero que el ejercicio físico está de acuerdo con mis valores | 0 | 1 | 2 | 3 | 4 |
| 21. Porque me pongo nervioso si no hago ejercicio regularmente | 0 | 1 | 2 | 3 | 4 |
| 22. Porque me resulta placentero y satisfactorio el hacer ejercicio | 0 | 1 | 2 | 3 | 4 |
| 23. Pienso que hacer ejercicio es una pérdida de tiempo | 0 | 1 | 2 | 3 | 4 |

**CUESTIONARIOS EN EL CONTEXTO DE LA AF EXTRACURRICULAR**

## 13. Intención de hacer actividad física en las próximas 5 semanas durante tu tiempo libre (fuera del horario escolar)

1 = totalmente desacuerdo; 7 = totalmente de acuerdo

| | | | | | | | |
|---|---|---|---|---|---|---|---|
| 1. **Tengo intención** de practicar deporte o actividad física durante mi tiempo libre en las próximas 5 semanas | 1 | 2 | 3 | 4 | 5 | 6 | 7 |
| 2. **Tengo pensado** hacer deporte o actividad física durante mi tiempo libre en las próximas 5 semanas | 1 | 2 | 3 | 4 | 5 | 6 | 7 |
| 3. **Espero poder** hacer deporte y actividad física durante mi tiempo libre en las próximas 5 semanas | 1 | 2 | 3 | 4 | 5 | 6 | 7 |

## 14. ¿Cuáles son las barreras o motivos por los que siempre o a veces no realizas actividad física en tu tiempo libre, fuera del instituto?

| | No es un problema | Es un pequeño problema | | | Es un gran problema | | |
|---|---|---|---|---|---|---|---|
| 1. No dispongo de un buen sitio para hacer actividad física | | 1 | 2 | 3 | 4 | 5 | 6 |
| **2.** No tengo a nadie con quien realizar actividad física | | 1 | 2 | 3 | 4 | 5 | 6 |
| **3.** No tengo el equipo necesario para hacer actividad física | | 1 | 2 | 3 | 4 | 5 | 6 |
| **4.** No es seguro estar jugando en la calle, parque, patio... | | 1 | 2 | 3 | 4 | 5 | 6 |
| **5.** No tengo suficiente tiempo | | 1 | 2 | 3 | 4 | 5 | 6 |
| 6. Tengo demasiados deberes en el instituto | | 1 | 2 | 3 | 4 | 5 | 6 |
| **7.** Tengo muchas tareas que realizar en casa | | 1 | 2 | 3 | 4 | 5 | 6 |
| **8.** Tengo que estudiar mucho | | 1 | 2 | 3 | 4 | 5 | 6 |
| **9.** No me gusta la actividad física | | 1 | 2 | 3 | 4 | 5 | 6 |
| **10.** Por lo general estoy cansado/a para hacer actividad física | | 1 | 2 | 3 | 4 | 5 | 6 |
| 11. No disfruto de la actividad física | | 1 | 2 | 3 | 4 | 5 | 6 |
| **12.** No soy bueno/a en las actividades físicas y/o deportivas | | 1 | 2 | 3 | 4 | 5 | 6 |
| **13.** Me da miedo lesionarme | | 1 | 2 | 3 | 4 | 5 | 6 |
| **14.** Soy muy perezoso/a para hacer actividad física | | 1 | 2 | 3 | 4 | 5 | 6 |
| **15.** Hacer actividad física es aburrido | | 1 | 2 | 3 | 4 | 5 | 6 |
| **16.** Me pongo nervioso/a cuando hago actividad física | | 1 | 2 | 3 | 4 | 5 | 6 |

**Indica otras razones por las que no haces actividad física durante tu tiempo libre fuera de la hora escolar:**

## 15. CUESTIONARIO INTERNACIONAL DE ACTIVIDAD FÍSICA

Estamos interesados en saber el tipo de actividad física que la gente hace como parte de su vida diaria. Las preguntas se referirán al tiempo que estuviste físicamente activo(a) en los **últimos 7 días**. Por favor, responde a cada pregunta, aunque no te consideres una persona activa. Por favor, piensa en aquellas actividades que forman parte de tu vida cotidiana y que sueles hacer en el instituto, en casa, para ir de un sitio a otro y en tu tiempo libre de descanso, ejercicio o deporte.

Piensa en todas aquellas actividades **VIGOROSAS** que realizaste en los **últimos 7 días**. Actividades **vigorosas** son las que requieren un esfuerzo físico fuerte e intenso y te hacen respirar mucho más rápido de lo normal. Piensa solamente en las actividades que hiciste por lo menos durante 10 minutos continuos.

1. Durante los **últimos 7 días**, ¿Cuántos días realizaste actividades físicas **VIGOROSAS** como correr, jugar al fútbol o pedalear rápido en bicicleta?

_______ **días por semana**

☐ Ninguna actividad física vigorosa ➡ **Pasa a la pregunta 3**

2. ¿Cuánto tiempo te llevó realizar actividades físicas **VIGOROSAS** en cada día que las practicaste?

| | L | M | X | J | V | S | D |
|---|---|---|---|---|---|---|---|
| horas por día | | | | | | | |
| minutos por día | | | | | | | |

☐ No lo sabes/No estás seguro(a)

Piensa en todas aquellas actividades **MODERADAS** que realizaste en los **últimos 7 días**. Actividades **moderadas** son aquéllas que requieren un esfuerzo físico moderado y te hacen respirar algo más rápido de lo normal. Piensa solamente en esas actividades que hiciste por lo menos durante 10 minutos continuos.

3. Durante los **últimos 7 días**, ¿Cuántos días hiciste actividades físicas **MODERADAS** tales como danza, nadar o jugar a dobles un partido de tenis? No incluyas caminatas.

_____ **días por semana**

☐ Ninguna actividad física moderada ➡ **Pasa a la pregunta 5**

4. En los **últimos 7 días**, ¿Cuánto tiempo dedicaste a hacer actividad física **MODERADA** para cada día?

| | L | M | X | J | V | S | D |
|---|---|---|---|---|---|---|---|
| horas por día | | | | | | | |
| minutos por día | | | | | | | |

☐ No lo sabes/No estás seguro(a)

Piensa en el tiempo que dedicaste a **CAMINAR** en **los últimos 7 días**. Esto incluye **caminar** en el instituto o en casa, para desplazarte de un lugar a otro, o cualquier otra caminata que podrías hacer en el tiempo de ocio.

**5. Durante los *últimos 7 días*, ¿en cuántos *caminaste* por lo menos *10 minutos* seguidos?**

**_______ días por semana**

☐ Ninguna caminata ➡ **Pasa a la pregunta 7**

**6. ¿Cuánto tiempo en total dedicaste a CAMINAR en cada día?**

| | L | M | X | J | V | S | D |
|---|---|---|---|---|---|---|---|
| horas por día | | | | | | | |
| minutos por día | | | | | | | |

☐ No lo sabes/No estás seguro(a)

La última pregunta es acerca del tiempo que pasaste **SENTADA/O** durante los **últimos 7 días**. Esto incluye el tiempo en el instituto, en casa y durante el tiempo libre. Puedes incluir el tiempo que pasaste sentado en el escritorio, visitando amigos, leyendo, o mirando la televisión.

**7.** Durante los **últimos 7 días**, **¿Cuánto tiempo pasaste *SENTADA*/O?**

| | L | M | X | J | V | S | D |
|---|---|---|---|---|---|---|---|
| horas por día | | | | | | | |
| minutos por día | | | | | | | |

☐ No lo sabes/No estás
